# COVID-19, Pre-Eclampsia, and Complement System

**DOI:** 10.3389/fimmu.2021.775168

**Published:** 2021-11-17

**Authors:** Chiara Agostinis, Alessandro Mangogna, Andrea Balduit, Azin Aghamajidi, Giuseppe Ricci, Uday Kishore, Roberta Bulla

**Affiliations:** ^1^ Institute for Maternal and Child Health, Istituto di Ricovero e Cura a Carattere Scientifico (IRCCS) Burlo Garofolo, Trieste, Italy; ^2^ Department of Life Sciences, University of Trieste, Trieste, Italy; ^3^ Department of Immunology, School of Medicine, Iran University of Medical Sciences, Tehran, Iran; ^4^ Department of Medical, Surgical and Health Science, University of Trieste, Trieste, Italy; ^5^ Biosciences, College of Health, Medicine and Life Sciences, Brunel University London, Uxbridge, United Kingdom

**Keywords:** COVID-19, complement system, SARS-CoV-2, pregnancy, pre-eclampsia

## Abstract

COVID-19 is characterized by virus-induced injury leading to multi-organ failure, together with inflammatory reaction, endothelial cell (EC) injury, and prothrombotic coagulopathy with thrombotic events. Complement system (C) *via* its cross-talk with the contact and coagulation systems contributes significantly to the severity and pathological consequences due to SARS-CoV-2 infection. These immunopathological mechanisms overlap in COVID-19 and pre-eclampsia (PE). Thus, mothers contracting SARS-CoV-2 infection during pregnancy are more vulnerable to developing PE. SARS-CoV-2 infection of ECs, *via* its receptor ACE2 and co-receptor TMPRSS2, can provoke endothelial dysfunction and disruption of vascular integrity, causing hyperinflammation and hypercoagulability. This is aggravated by bradykinin increase due to inhibition of ACE2 activity by the virus. C is important for the progression of normal pregnancy, and its dysregulation can impact in the form of PE-like syndrome as a consequence of SARS-CoV-2 infection. Thus, there is also an overlap between treatment regimens of COVID-19 and PE. C inhibitors, especially those targeting C3 or MASP-2, are exciting options for treating COVID-19 and consequent PE. In this review, we examine the role of C, contact and coagulation systems as well as endothelial hyperactivation with respect to SARS-CoV-2 infection during pregnancy and likely development of PE.

## Introduction

The first case of severe acute respiratory syndrome coronavirus 2 (SARS-CoV-2) infection, responsible for the coronavirus disease 2019 (COVID-19) outbreak, was reported in the Chinese town of Wuhan in the late 2019 (1). The emerging coronavirus spread worldwide over the following months has been officially recognized as a global pandemic since 11^th^ March 2020 (2).

SARS-CoV-2 resembles several characteristics and pathways of infection with other two members of the *Coronaviridae* family: the SARS pandemic in 2002 and the Middle East respiratory syndrome (MERS) in 2012, with a fatality rate of 10% and 36%, respectively (3). CoVs encompass a group of enveloped and single-stranded RNA viruses identified in birds and mammals, and can cause gastrointestinal, central nervous system, and respiratory tract infections (4). The main structural proteins, encoded by specific genes in open reading frame (ORF)-1 downstream regions, include Spike (S), Envelope (E), Membrane (M), and Nucleocapsid (N), S protein being responsible for SARS-CoV-2 invasion of the host cells (5, 6). Among the different receptors identified as cellular entry mediators, the main target receptor of the SARS-CoV-2 virus is the angiotensin-converting enzyme 2 (ACE2) (7), which plays an important role in the renin-angiotensin-aldosterone system (RAAS) for the regulation of blood pressure and electrolyte homeostasis (8), and is widely expressed by most tissues, accounting for the high tropism of the virus. SARS-CoV-2 cell entry also depends on the cellular transmembrane serine protease 2 (TMPRSS2), responsible for S protein priming (7). Extracellular matrix metalloproteinase inducer (EMMPRIN, also known as basigin or CD147), a cell surface glycoprotein that belongs to the immunoglobulin superfamily and activates metalloproteases, has been regarded as a target for SARS-CoV-2 attachment and entry into the host cell (9, 10). In addition, the receptor for Semaphorin-3, named neuropilin-1, has also been demonstrated to facilitate SARS-CoV-2 infection (11).

The SARS-CoV-2 infection damages various organs *via* different pathogenic mechanisms, including direct viral damage to the host cells/tissues through pneumocyte syncytia formation (12) and Golgi apparatus rupture (13), RAAS disruption and endothelial cell (EC) damage resulting in inflammation, endotheliitis and thrombosis (14). Moreover, an unfavorable dysregulation of immune response characterized by lymphopenia and cytokine storm has been reported as a key pathogenic mechanism in COVID-19 (15, 16). In this context, the complement system (C) primary role as a first line of defense against infectious agents would suggest for a protective function in enhancing virus neutralization and phagocytosis. However, C activation has been proposed as a contributor in disease progression to a more severe and lethal stage, which shares important pathophysiological features, in particular endothelial damage, with a pregnancy disorder called pre-eclampsia (PE) (17, 18). This review aims to shed light on the role of the C in pregnant women with COVID-19 developing PE.

## The Complement System

**Figure 1:**
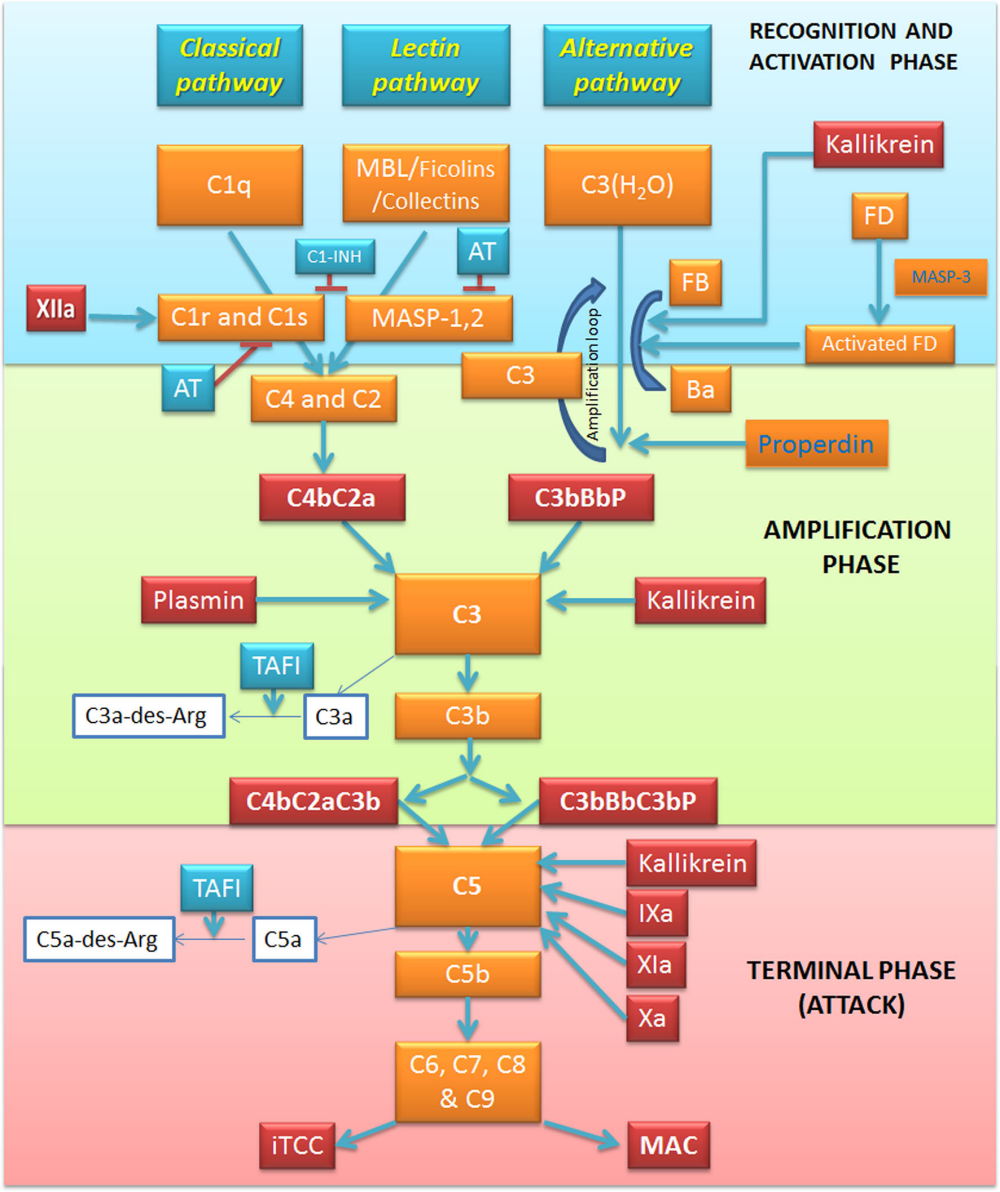
Overview of the complement system (C) and its interplay with the coagulation and kallikrein-kinin pathways. The activation of C *via* three pathways: classical, alternative and lectin. The recognition and activation phases in each pathway converge on the formation of C3 convertase. The amplification phase is initiated when the C3 convertase cleaves C3, and C3b attachment to C3 convertases changes their substrate specificity, allowing them to become C5 convertase. The C5 convertase cleaves C5, starting the last phase (Terminal-Attack phase), in which C5b forms the membrane-attack complex with C6, C7, C8, and C9, resulting in cell perturbation. Kallikrein exerts its activity on C activation in all these three phases. Factor (F) XIIa activates the classical pathway, whereas plasmin enhances the amplification loop. Thrombin, FIXa, FXa, FXIa, and kallikrein directly activate C5. AT, Antithrombin; C1-INH, C1-inhibitor; F, Factor; MAC, Membrane attack complex; MASP, MBL associated serine protease; MBL, Mannose binding lectin; iTCC, Inactive terminal complement complex; TAFI, Thrombin activatable fibrinolysis inhibitor.

The C, as a powerful arm of the innate immunity, has a pivotal role in the recognition of potential danger signals and in the clearance of pathogens, apoptotic and necrotic cells (19). The C acts as a functional bridge between innate and adaptive immunity, being a system that “complements” the function of antibodies in the modulation of an integrated host defense (20). The C is comprised of over 50 plasma and cell surface proteins, including activation effectors, regulators and cell surface receptors (21). These proteins are organized to take part in three independent but interactive activation pathways (**Figure 1**): classical, lectin and alternative, converging on the common activation of the major component C3 and in the production of proinflammatory mediators, opsonization, membrane attack complex (MAC) formation, and target cell lysis (22).

### Classical Pathway

The classical pathway is mainly triggered by the interaction between the C1 and immune complexes, apoptotic and necrotic cells (23). C1 complex consists of three sub-components: the classical pathway recognition molecule C1q and the serine proteases C1r and C1s. The activation process is initiated by C1q recognition of the Fc domain of an array of IgM or IgG bound to an antigen. The consequent C1q conformational change is responsible for the activation of C1r serine-protease activity (24), which, in turn, acts as a trigger to the proteolytic activity of C1s, inducing C4 and C2 cleavage into two active components (C4b and C2a) and two small fragments released in the fluid phase (C4a and C2b) (25). C4b is able to covalently bind to membrane proteins and carbohydrates (26), and non-covalently to C2a inducing the C4b2a complex formation, also known as C3 convertase of the classical pathway (25). C3 convertase, attached to the target surface, is responsible for C3 cleavage into C3b and C3a anaphylatoxin (**Figure 1**).

### Lectin Pathway

The lectin pathway is an antibody-independent route in which mannan-binding lectin (MBL), ficolins (ficolin-1, -2 and -3) or collectin-11 act as pattern-recognition molecules through their binding to mannose residues and other carbohydrate ligands in the form of pathogen-associated molecular patterns (PAMPs), or damage-associated molecular patterns (DAMPs) (27). This binding triggers MBL interaction with MBL-associated serine proteases (MASPs), a family of serine proteases including three enzymatic proteins (MASP-1, MASP-2 and MASP-3) and two non-enzymatic factors (Map19 and Map44). MASP-3 is mainly involved in the alternative pathway activation (28), whilst MASP-2 activation by MASP-1, forming a dimer, activate the lectin pathway *via* C4 and C2 cleavage and C3 convertase formation (29).

### Alternative Pathway

The initiation of the alternative pathway is independent of immune complexes, being constitutively active at basal levels in the so-called “tick-over” mechanism and assuring a rapid and robust C activation in the presence of pathogens (30). Under normal physiological conditions, C3 undergoes constant low-grade activation by spontaneous hydrolysis, producing the C3(H_2_O) molecule, which is rapidly inactivated in the circulation (31). After its binding to C3(H_2_O), the plasma protein factor B (FB) is cleaved by the serine protease factor D (FD), losing the small fragment Ba, whilst the residual fragment Bb remains bound to C3(H_2_O) forming the fluid phase C3 convertase, C3(H_2_O)Bb. C3(H_2_O)Bb has the ability to cleave large amounts of C3 molecules into C3a and C3b. C3b is partly inactivated by hydrolysis; however, the interaction of C3b with surface components of microbial agents and damaged host cells can accelerate the alternative pathway and induce the association with FB, further cleaved by FD, and generation of the amplification loop convertase C3bBb. The rapid amplification loop is also boosted by the C3b molecules generated by either the classical or lectin pathway. The alternative pathway C3 convertases are highly labile, so they need to be stabilized by an up-regulator called factor P or properdin, increasing its half-life by 10-fold (32).

### Membrane Attack Complex Formation

All the three C activation pathways converge on the common C3 convertase formation and C3 cleavage into the anaphylatoxin C3a and the opsonin C3b (19, 33). The incorporation of the C3b fragment in the C3 convertase gives rise to the production of C5 convertase, which in turn, cleaves C5 to yield C5b and the anaphylatoxin C5a. Then, C5b sequentially interacts with the terminal C components C6, C7, C8, and C9, resulting in the formation of the C5b-9 terminal C complex (TCC). If TCC is fully inserted into a cell plasma membrane, it is called the MAC. MAC-mediated cell death can release DAMPs, which can result in further C activation. In many clinical conditions associated with massive C activation, in plasma or other fluids, the formation of a cytolytically inactive TCC (iTCC)' also called soluble C5b-9 (sC5b-9) complex, is helpful in several pro-inflammatory responses acting directly on endothelium (34).

### Main Regulators of Complement Activation

Abnormal C activation can be responsible for severe inflammatory conditions, as hereditary angioedema, paroxysmal nocturnal haemoglobinuria, and haemolytic uremic syndrome (25, 35). Several cell membrane-bound or soluble C regulators exist to control this system. C1-inhibitor (C1-INH) inhibits both classical and lectin pathway initiation through its action on C1r, C1s and MASP-2, disassembling C1 or ficolin/MBL-MASP complexes' respectively (36, 37). Moreover, C1-INH is also able to downregulate the alternative pathway convertase by interacting with C3b and inhibiting its binding to FB (38). At the C3 convertase level, the main regulator is FI (39)' a serine protease responsible for C3b and C4b cleavage. It needs several cofactors for enhancing its activity. C4b-binding protein (C4bp) represents the main cofactor of FI for C4b cleavage (40). Another cofactor of FI is FH that can bind to polyanionic molecules exposed on the membrane such as glycosaminoglycans, heparin and sialic acid. FH interacts with C3b promoting its hydrolysis by FI (39). In addition, the membrane-bound regulatory proteins, C Receptor 1 (CR1, CD35) (41) and Decay Accelerating Factor (DAF; CD55) (42), participate in C3b and C4b degradation. Membrane Cofactor Protein (MCP; also called CD46) (43) is involved in the acceleration of their decay of the C3- and C5-convertases. CD59 (also known as protectin), by binding to C5b-8, limits the incorporation of the C9 molecules, and consequently, formation of the MAC (44). If the cell is protected from lysis by CD59 (45), the sublytic attack can induce the release of inflammatory mediators (46, 47). When C5b-7, C5b-8 or C5b-9 assemble in plasma, the binding of the plasma proteins' vitronectin (alternative names S-protein) (48) and clusterin (49) (also known as SP40.40)' can lead to iTCC formation, preventing insertion into lipid bilayers and MAC formation.

## Role of Complement System in Viral Infections Including SARS-CoV-2

**Figure 2:**
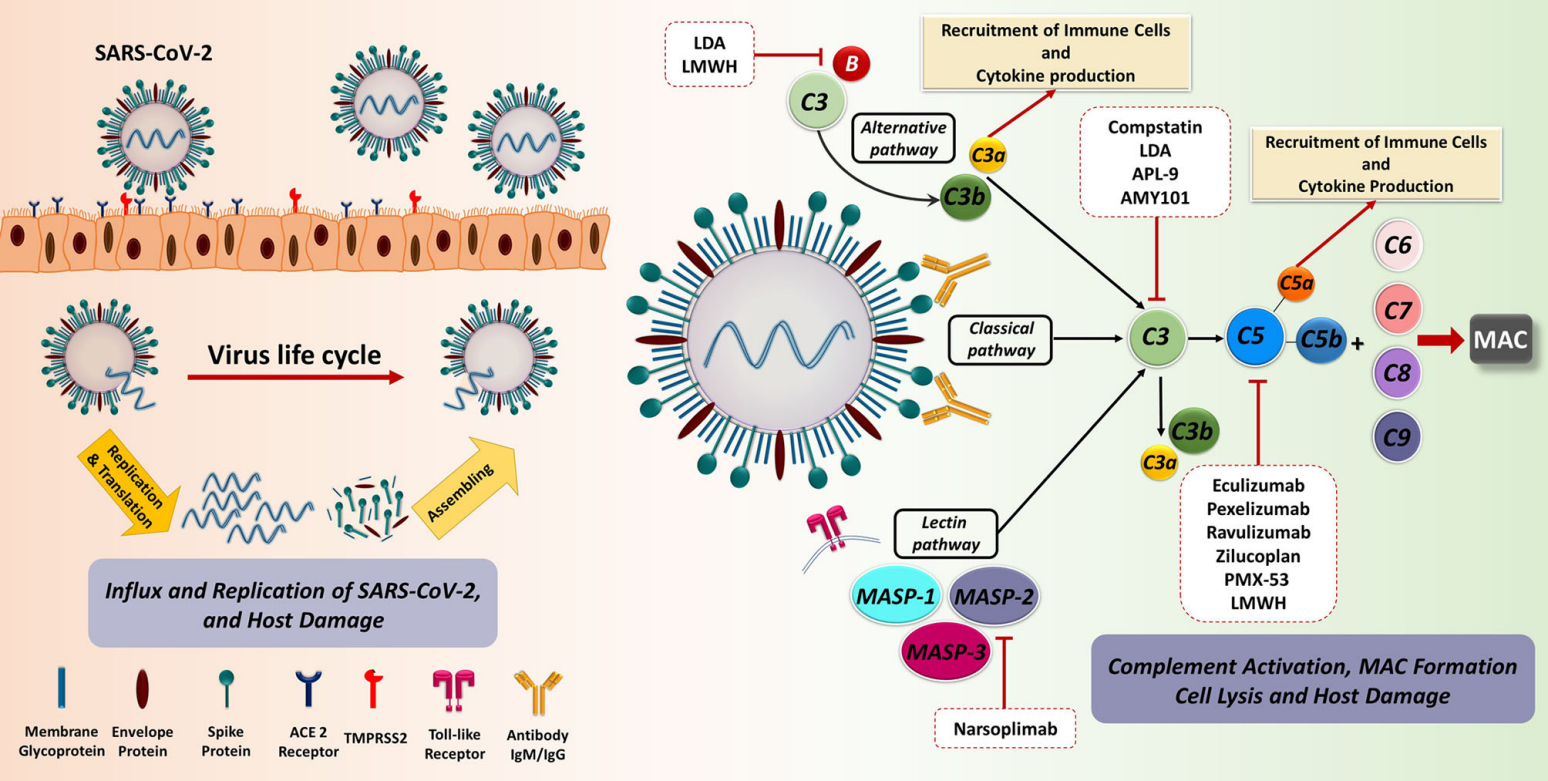
The recognition and binding of S protein to the ACE2 receptor are fundamental key events in SARS-CoV-2 invasion of the host cells. The engagement of ACE2 receptor and S protein of SARS-CoV-2 induces the fusion of virus and target cell. This process is orchestrated by the cleavage of S protein into S1 and S2 subunits *via* transmembrane serine protease priming known as TMPRSS. Subsequently, prompt viral replication occurs inside the infected cell, contributing to the dissemination of infection. The C as a first line of innate immune defence could recognize the virus. SARS-CoV-2 is capable of directly activating the C through the classical, alternative, and especially lectin pathways. Dysregulated C activation during the SARS-CoV-2 infection can cause serious damage to different organs. The blockade of C effectors has a potential therapeutic effect through the prevention of the recruitment of immune cell as well as immune activation. ACE2, Angiotensin-converting enzyme 2; LDA, Low-dose aspirin; LMWH, Low-molecular weight heparin; MAC, Membrane attack complex; MASP, MBL (mannose binding lectin) associated serine protease; SARS-CoV-2, Severe acute respiratory syndrome coronavirus 2; TMPRSS2, Transmembrane serine protease 2.

Acting in concert, the three pathways of the C cascade are effective in targeting both cell-free viral particles and virus-infected cells, boosting the anti-viral innate and adaptive immune responses (50). The antiviral activity of the C system usually takes place through four different, but not exclusive, mechanisms of action: C deposition and opsonization, MAC formation and viral cell lysis, production of pro-inflammatory anaphylatoxins, as well as enhancement of adaptive immunity (**Figure 2**) (51). The ‘eat me’ signal by C1q, C3b, and C4b components on the non-self-agents prompts opsonization and enhanced phagocytosis; this process can progress to MAC assembly and viral envelope lysis. Moreover, C3a and C5a anaphylatoxins are able to recruit neutrophils, mast cells, monocytes, macrophages, basophils, eosinophils, T and B cells, giving an important contribution to chemotaxis, NETosis, degranulation, production of cytokines, inflammation' and reactive oxygen species (ROS) production (52–54).

The most striking evidence supporting the importance of C in viral infection outcome is provided by the evolution of specific evasion mechanisms employed by viruses for subverting C (55). First of all, viruses can recruit and exploit soluble and membrane-bound host C regulators, as well as encode their own C regulatory proteins (56). Moreover, they are also capable of using C regulators and receptors for cellular entry or even to modulate C protein expression through the upregulation of C regulators or the downregulation of C activators (57).

It is now widely accepted that, during the initial stages of the infection, SARS-CoV-2 proteins are able to directly activate all three pathways of C (58): N protein is able to induce the MASP-2-mediated activation of lectin pathway (59) and S protein is responsible for the alternative pathway activation (60), whilst classical pathway is usually activated at advanced stages *via* immune complexes and C-reactive protein involvement (61, 62). Evidence so far suggests that C may be beneficial in the early stages of SARS-CoV-2 infection due to its participation in virus elimination; however, C activation may be severely harmful in later phases.

In COVID-19, the initial viral invasion phase is usually followed by an immunopathological phase, which is characterized by an uncontrolled immunological response causing pulmonary, and sometimes, systemic inflammation, in which C is also involved. In particular, recent findings indicate that excessive or deregulated C activation may occur in COVID-19 patients (62, 63), triggering anaphylatoxin generation and binding to their receptors (64), with subsequent inflammatory cell recruitment in the lungs and other organs (65). This contributes to cytokine storm, EC injury, intravascular coagulation and thrombosis (66, 67).

## Endotheliitis, Complement System, and Pathogenesis of COVID-19

**Figure 3:**
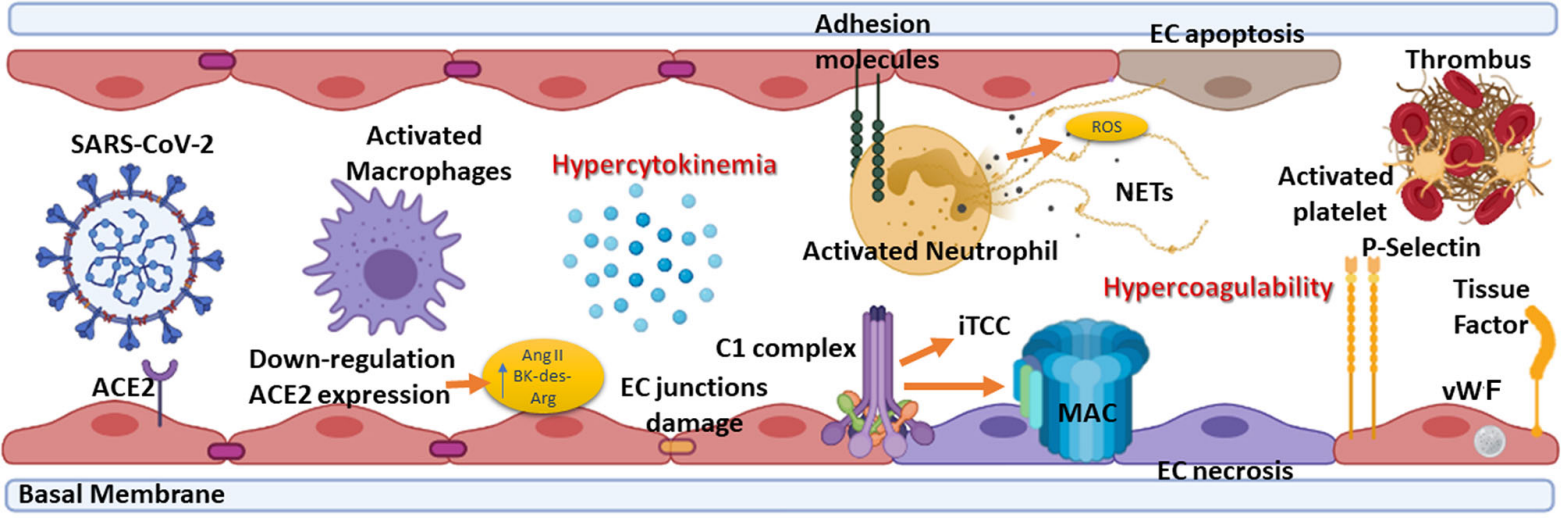
Summary of the elements involved in endotheliitis. COVID-19 pathogenesis is characterized by an initial virus-induced injury leading to multi-organ failure, coupled with an intense inflammatory reaction, endothelial cell (EC) injury, and a prothrombotic coagulopathy with thrombotic events. The hypercytokinemia induced by severe acute respiratory syndrome coronavirus 2 (SARS-CoV-2) causes the expression by ECs of adhesion molecules such as intercellular adhesion molecule 1 (ICAM-1), vascular cell adhesion molecule 1 (VCAM-1) and P-selectin. Neutrophils are recruited and contribute to this process through the release of neutrophil extracellular traps (NETs), which directly activate factor XII and, thus, the contact-dependent pathway of coagulation. NETs also bind von Willebrand factor (vWF) and help to recruit platelets. The feed-forward loop is amplified by the effect of P-selectin, vWF, and fibrinogen expression by ECs in response to hypercytokinemia, causing platelets to directly bind to ECs leading to their activation and hypercontractility, which can lead to disruption of cell-cell junctions and vascular leakage. ACE2, Angiotensin-converting enzyme 2; Ang II, Angiotensin II; BK, Bradykinin; MAC, Membrane attack complex; iTCC, Inactive terminal complement complex; ROS, Reactive oxygen species.

COVID-19 pathogenesis is characterized by an initial virus-induced injury and consequent multi-organ failure, coupled with an intense inflammatory reaction, EC injury, and a prothrombotic coagulopathy with thrombotic events. The progression from mild to severe COVID-19 is characterized by the transition from an epithelial to an endothelial disease (14, 68). Indeed, ECs, playing a pivotal role in the regulation of immune response, initiation, and maintenance of inflammatory process (69), coagulation, and platelet function, are key players in various pathological manifestations associated with COVID-19 (**Figure 3**) (70, 71).

Direct viral infection of ECs, *via* SARS-CoV-2 receptors, ACE2 and TMPRSS2, present on their surface (7), is able to provoke endothelial dysfunction and disruption of vascular integrity, leading to hyperinflammation and hypercoagulability (72). The binding of SARS-CoV-2 to ACE2 hampers its enzymatic activity, with consequent enhanced vascular permeability (72) associated with activation of kallikrein-kinin system and bradykinin (BK) accumulation (7, 73). Furthermore, reduced ACE2 expression by binding of SARS-CoV-2 on ECs limits the degradation of des-Arg^9^-BK, the active metabolite of BK, into inactive peptides, increasing prothrombotic signaling *via* the activation of BK receptor 1, expressed during inflammatory conditions (72, 74). ACE2 downregulation determines angiotensin II accumulation, which enhances vascular permeability through AT1 receptor and promotes tissue damage, but also reduces Mas activation by angiotensin 1-7, supporting a local pro-inflammatory and pro-thrombotic EC phenotype (75, 76).

Not only a direct virus-dependent effect on ECs has been observed, but also host-specific factors seem to contribute to systemic endothelial dysfunction in COVID-19. The C seems primarily involved in this process, the activation of its three pathways and MAC formation as contributors to EC swelling and even disruption (77). Indeed, in severe disease conditions, elevated levels of serum MAC (62), as well as a strong immunohistochemical staining for deposited C5b-9 in the microvasculature, have been detected (61) in co-localization studies with SARS-CoV-2 N protein (62, 78). MBL, MASP-2, C4a and C3 deposits have also been observed (78).

Immune complexes comprising SARS-CoV-2 specific antibodies and viral antigens may lead to EC injury through the activation of C1 complex of the classical pathway and induction of antibody-dependent cytotoxicity. Increased levels of C3a and C5a, due to C hyperactivation, amplify the vicious cycle of vascular integrity disruption, promoting infiltration of neutrophils which potentiates ROS production, degranulation and NETosis, ultimately provoking further injury to ECs (79). Elevated serum levels of C5a, the most potent C anaphylatoxin, have also been reported in severe COVID-19 patients, whereas circulating C5a levels in patients with mild manifestations are similar to those of the healthy controls (62). Furthermore, a close association of C5a-C5aR axis with inflammation and endotheliitis has been observed in the pathogenesis of severe COVID-19 (64).

EC damage, characterized by disruption of cell-cell junctions and vascular leakage, exposes basement membrane to circulatory platelets, initiating platelet aggregation, hypercoagulable state and thrombotic events (72) (**Figure 3**), which are frequently observed in severe COVID-19 patients (80).

## Complement System and Its Role in the Thrombotic Events in COVID-19

The C activation has been associated with COVID-19-related coagulopathy and thromboembolia (81), suggesting an interplay between C and coagulation system (**Figure 1**). Interestingly, SARS-CoV-2 infection is able to induce the transcription of C (C1r, C1s, factor B and C3) and coagulation genes (fibrinogen) in pneumocytes and hepatocytes (81).

**Figure 4:**
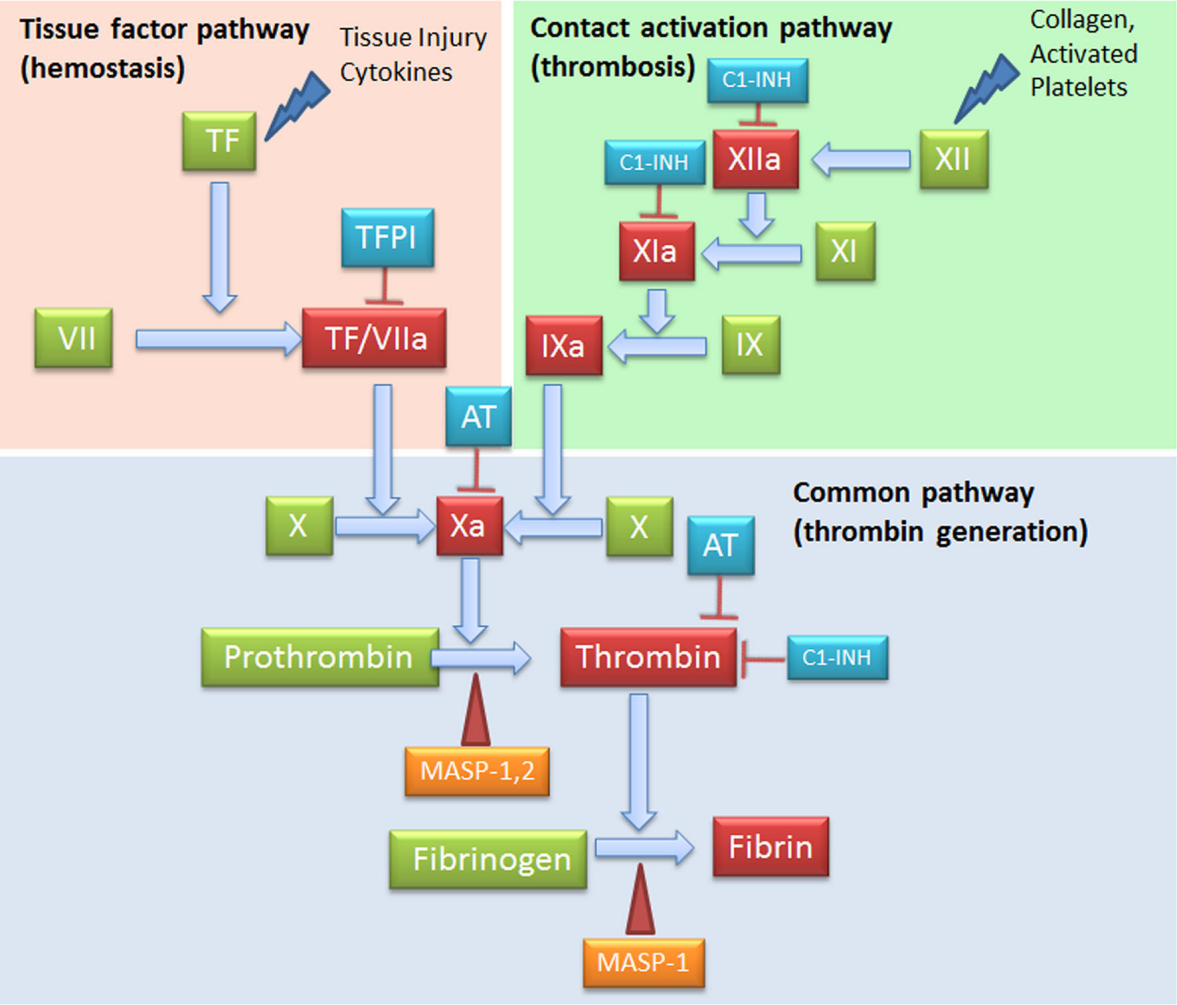
Complement Components that act on the coagulation system. The intrinsic (FXII) and the extrinsic (TF/thromboplastin) pathways initiate the coagulation cascade, both converging at the common point of FX activation. FXa is causes prothrombin (FII) activation in thrombin (FIIa), which leads to the formation of fibrin from the soluble fibrinogen. MASP-1 and MASP-2 directly activate thrombin by cleaving prothrombin, and MASP-1 is able to cleave fibrinogen to generate fibrin monomers. C1-INH exerts its inhibitory activity on the coagulation system by acting on FXIIa, FXIa and thrombin. AT, Antithrombin; C1-INH, C1-inhibitor; MASP, MBL (mannose binding lectin) associated serine protease; TF, Tissue factor; TFPI, Tissue factor pathway inhibitor.

C and coagulation systems are evolutionarily linked in terms of functional similarities and shared structural motifs (82). The process of coagulation is activated through the contact (intrinsic) and the Tissue Factor (TF) (extrinsic) pathways, both converging on factor (F) X activation. The intrinsic pathway starts from FXII activation by negatively charged surfaces, such as phospholipids present on activated platelets and exposed subendothelial collagen (83) (**Figure 4**). The extrinsic pathway is responsible for a quick and efficient *in vivo* hemostasis, once activated by TF release by damaged cells or expression on the surface of activated monocytes, ECs, and other non-vascular cells. TF then converts FVII to FVIIa (82). The common factor X, upon activation, is then responsible for prothrombin (FII) activation in thrombin (FIIa), which in turn, triggers the formation of fibrin from the soluble fibrinogen (84) (**Figure 4**).

**Figure 5:**
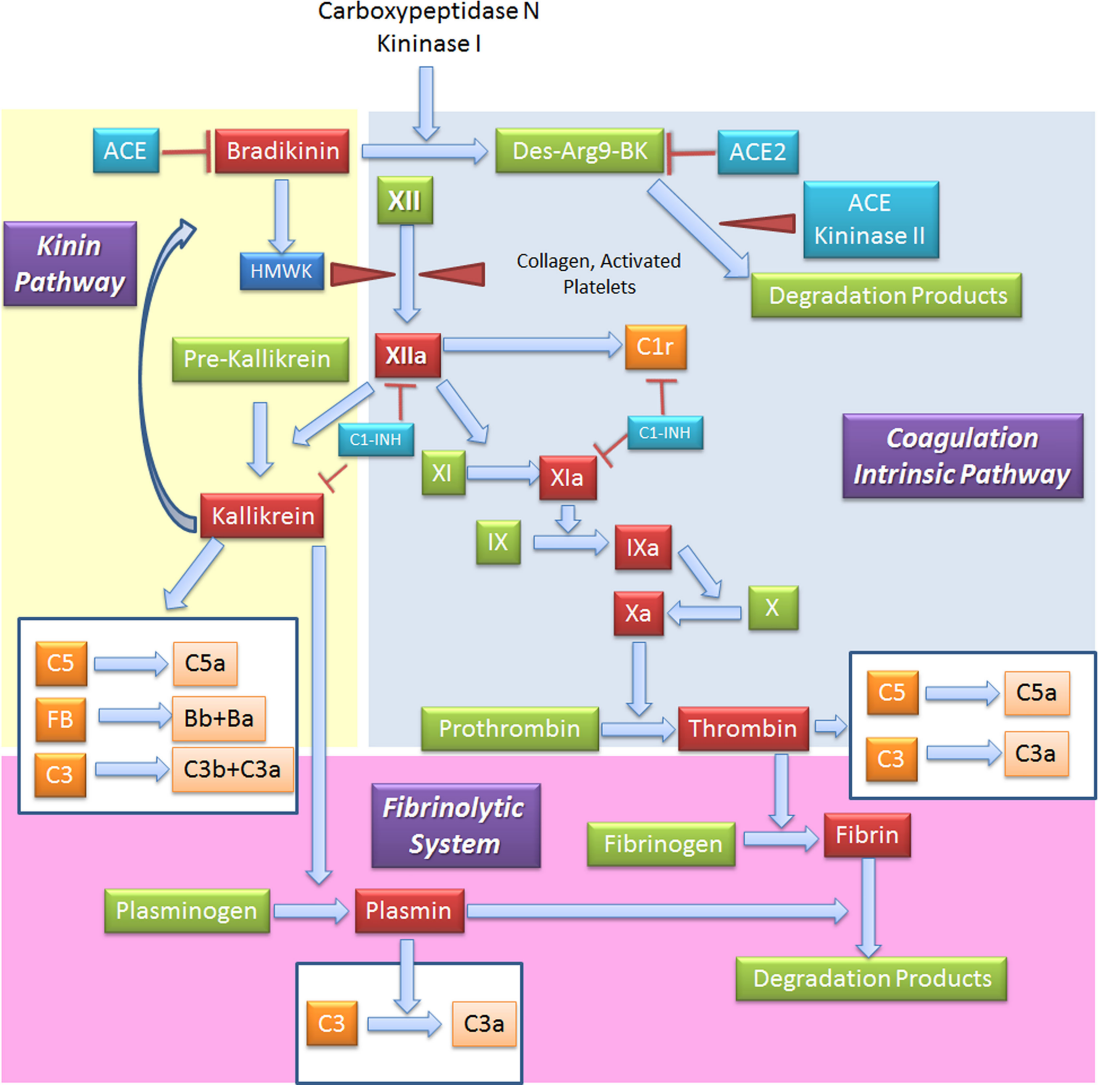
Interplay between coagulation, fibrinolytic, contact and complement systems. The kinin system is comprised of proteins that participate in the coagulation and inflammation. In the kinin/contact system activation may occur *via* auto-activation of FXII in contact with negatively charged surfaces, or the plasma kallikrein-kinin system, as activation in plasma may be driven by pre-kallikrein activation. Kinins are vasoactive peptides produced by the action of a specific protease, called plasma kallikrein on kininogens. Plasma kallikrein, through the cleavage of the plasma glycoprotein precursor high-molecular-weight kininogen (HMWK), produces bradykinin. Bradykinin (BK) causes increase in vascular permeability, contraction of smooth muscle, dilation of blood vessels, and pain when injected into the skin, with similar effects to histamine. It exerts profibrinolytic properties by stimulating release of tissue plasminogen activator from endothelial cells. BK is quickly inactivated by redundant membrane and soluble kininase. The coagulation intrinsic pathway of the coagulation system is composed by plasma proteins activated by FXII, a protein synthesized by the liver that can be activated by collagens, basal membrane and activated platelets. The coagulation system culminates in the formation of thrombin (FIIa) from prothrombin (FII) and in the formation of fibrin from the soluble fibrinogen. When generated, FXIIa is also responsible of the activation of other three systems: (a) C, *via* activation of C1r and C1s; (b) inflammatory kallikrein-kinin pathway by converting pre-kallikrein into active plasma kallikrein, inducing the cleavage of both FXII into FXIIa and high molecular weight kininogen to BK; and (c) fibrinolytic system by plasma kallikrein activation, in turn cleaving plasminogen into plasmin, an enzyme that degrades fibrin clots. Several coagulation system enzymes, including thrombin, FIXa, FXa, FXIa, and kallikrein, directly activate C5. Kallikrein also activates C3 and factor. ACE, Angiotensin-converting enzyme; C1-INH, C1-inhibitor; FB, Factor B.

When generated, FXIIa is also responsible of the C activation *via* C1r and C1s (85), and that of the inflammatory kallikrein-kinin pathway by converting pre-kallikrein into active plasma kallikrein, which cleaves FXII into FXIIa and high molecular weight kininogen to BK and the fibrinolytic system by plasma kallikrein activation of pro-urokinase into urokinase, which in turn, cleaves plasminogen into plasmin, an enzyme that degrades fibrin clots (86, 87) (**Figure 5**).

The coagulation system is also involved in inflammation and tissue remodelling through the interaction of coagulation proteases with four distinct protease-activated receptors (PAR1, PAR2, PAR3 and PAR4) (83). The interplay between thrombosis and inflammation is ensured by PAR1 (previously known as the thrombin receptor) cleavage, upon thrombin-PAR1 interaction, resulting into an overall pro-inflammatory state characterized by the release of P-selectin, von Willebrand factor (vWF) and the disruption of the endothelial barrier function (88).

### Complement and Tissue Factor Expression

TF (also known also thromboplastin), a transmembrane receptor for FVII/VIIa, acts as initiator of the extrinsic coagulation pathway (89). Several C activation factors induce TF expression on leukocytes and ECs (90–92). C5a increases TF activity in the circulation (93). C5a, by interacting with C5aR, mediates the expression of TF in neutrophils enhancing their procoagulant activity (93) The TF expression in monocytes can also be induced by the membrane insertion of the C5b-7 (94). In addition, the terminal components of the C cascade stimulate the synthesis and release of TF (90); in fact, sublytic MAC (92) and iTCC (91) have been shown to stimulate the expression of TF on ECs, triggering a prothrombotic state through FVII-dependent activation of FX.

A C-linked release of TF has been observed in COVID-19, mainly due to a direct virus-dependent effect on ECs, in which viral infection of the endothelium provokes release of viral proteins able to activate C with consequent stimulation of TF production by neutrophils, monocytes and ECs, as well as causes endothelial injury that would expose subendothelial TF (66, 67, 95, 96).

### Complement and von Willebrand Factor Interaction

vWF is a complex multimeric plasma glycoprotein critical for normal haemostatic function; under physiological conditions, it is synthesized by ECs and stored in the Weibel–Palade bodies, and by megakaryocytes, being primarily stored in α-granules of platelets, as ultra-large vWF (97, 98). vWF has two main roles in haemostasis: firstly, to recruit and tether platelets at sites of vascular injury, facilitating aggregation; secondly, vWF acts as a protective carrier molecule for procoagulant FVIII. The assembly of C5b-9 on human ECs results in the secretion of high molecular weight multimers of vWF and release of membrane particles from the EC surface, which express binding sites for FVa, supporting prothrombinase activity (99, 100). Moreover, ultra-large vWF offers an activating surface for the assembly of the alternative pathway convertase; FH is able to reduce ultra-large vWF to smaller forms (101).

Plasma vWF levels are significantly increased in patients with COVID-19 (102), due to EC activation, thereby facilitating recruitment and aggregation of platelets (103) and tethering of leucocytes to the vessel wall (104). A C-linked release of vWF has been observed in response to sublytic MAC addition to ECs (99, 105).

### Complement and Thrombin Generation

MASPs associated with surface-bound MBL or ficolins exert their activity also in the coagulation process by cleaving coagulation factors, despite a slower kinetics as compared to coagulation proteases (27). MASP-1 and MASP-2 directly cleave prothrombin causing thrombin generation; MASP-1 is also able to cleave fibrinogen to generate fibrin monomers. Furthermore, MASP-1 is able to induce the formation of FXIIIa, a fibrin stabilizing factor, and that of thrombin-activatable fibrinolysis inhibitor (TAFI), an attenuator of the fibrinolytic rate. Among thrombin inhibitors, C1-INH and anti-thrombin III+heparin exert their inhibitory effect also on MASP-1 and MASP-2, whereas α2-macroglobulin does not abolish lectin pathway activation (37). The key TF Pathway Inhibitor (TFPI), expressed by microvascular ECs, can interfere with the lectin pathway by blocking MASP-2 (106). In addition to MASPs, sC5b-9 can also be involved in thrombin generation and in the flipping of EC and platelet phospholipid membranes, supporting prothrombinase assembly (FXa and FVa binding) (107).

It has been shown that thrombin can cleave C3 and C5, producing biologically active anaphylatoxins, C3a and C5a (108). Within the context of an overall increased thrombin generation, as observed in COVID-19, this mechanism accounts for the amplification of the feed-forward loop between C and coagulation, linking both cascades *via* multiple direct interactions.

### Reciprocal Regulation of Complement and Coagulation Pathways

Several C factors are able to control different steps of the coagulation pathway and vice versa (**Figures 1**, **5**). C1-INH can exert a dual inhibitory function on C and coagulation, by respectively inhibiting C1 complex and FXIIa, which is also responsible for C activation through C1r and C1s; regulation of contact system is also C1-INH-dependent through inactivation of plasma kallikrein (109). Furthermore, C1-INH is able to inhibit FXIa (110) and thrombin (111). Interestingly, the interaction of SARS-CoV proteins with C1-INH during viral infection determines C1-INH blockage (112, 113). Low C1-INH serum levels were shown as a predictive factor of progression to respiratory distress in COVID-19 (114).

Among coagulation regulators, an important cross-talk is represented by thrombomodulin (TM), a cell-bound regulator with anticoagulant properties, which can interact with FH enhancing its regulatory activity and consequently accelerating the degradation of C3b into inactive iC3b (115). Several coagulation system enzymes, such as thrombin, factor IXa, factor Xa, factor XIa, and kallikrein, are responsible for direct C5 activation. Kallikrein also activates C3 and factor B. In animal models of arterial and venous thrombosis, plasmin also exerts C5 convertase activity (116) (**Figure 5**). TAFI, also known as plasma carboxypeptidase B2 or R, suppresses fibrinolysis in physiological conditions. Following activation by thrombin/TM and/or plasmin, it can inactivate C3a and C5a, potentiating the action of the constitutive carboxypeptidase N as a supplementary inhibitor (117). Interestingly, markedly elevated circulating TAFI levels are reported in COVID-19 patients, being implicated in microvascular fibrin deposition (118).

### Complement-Platelet Crosstalk

Platelet activation is also responsible for the release of C components, including C1q, C3, C4, and C5b-9 (119, 120). C3a, C5a and, to a lesser extent, C4a, promote platelet aggregation and activation through their binding to cognate receptors C3aR or C5aR1 and C5aR2 (121, 122). Despite the absence of a specifically recognized receptor for C4a, it is able to bind to PAR1 and PAR4, participating in platelet activation. Activated platelets expose P-selectin, which is a receptor for C3b, providing a site for the assembly of the alternative pathway C3 convertase (123). Concurrently, C5a and the C5b-9 induce the expression of P-selectin and vWF by ECs (105), promoting platelet adhesion and aggregation, and release of TM from cell surface (124), triggering the coagulation cascade.

MAC is able to activate platelets and promote platelet aggregation (107, 125). The assembly of MAC on human platelets also results in a dose-dependent increase in the binding of FVa and FXa, which increases platelet prothrombinase activity.

## Pre-Eclampsia in COVID-19 and the Role of Complement System

PE is a frequent pregnancy-related disease (2-6% incidence in healthy nulliparous women), contributing to 20-25% of perinatal mortality, characterized by the onset of hypertension, proteinuria and multi-organ impairments. PE has been defined by the International Society for the Study of Hypertension in Pregnancy as the manifestation of arterial hypertension and proteinuria (300 mg/d) occurring after 20 weeks of gestation or as new-onset arterial hypertension combined with organ dysfunctions, such as renal failure, liver dysfunction, hematological or neurological abnormalities, intrauterine growth restriction, or uteroplacental insufficiency (126, 127). Approximately 10-20% of women with severe PE develop HELLP (hemolysis, elevated liver enzymes and low platelets) syndrome as a further complication (128).

**Figure 6:**
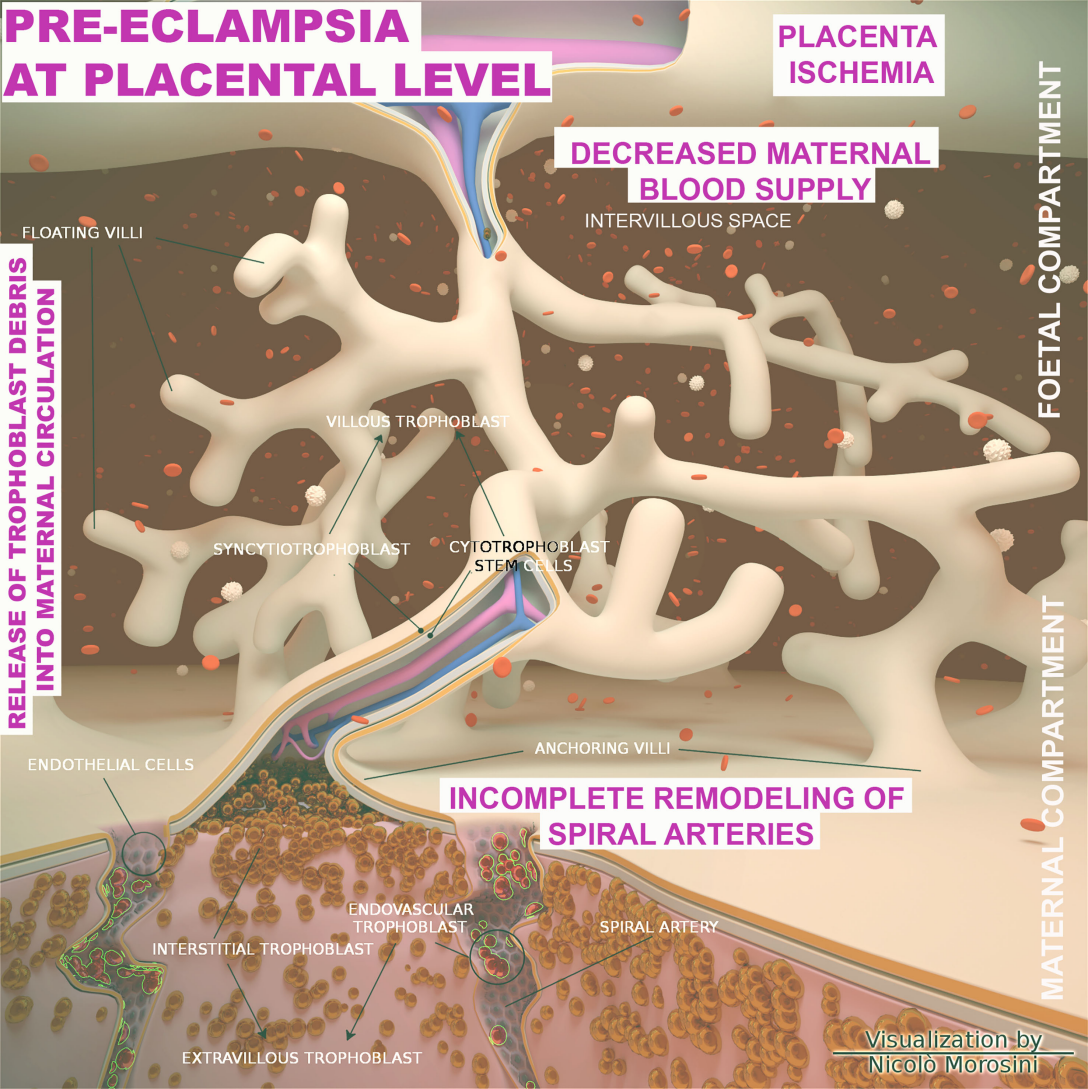
Schematic representation of the placenta architecture in pre-eclampsia (PE). Overview of the structural organization of the placenta and feto/maternal interface during the abnormal placentation in PE. The diagram was designed using the Blender 3D (Blender Foundation, Stichting Blender Foundation, Buikslotermeerplein, Amsterdam, the Netherlands). Edited with permission from Balduit et al. (133).

From an etiological perspective, PE is commonly distinct in early onset PE (EOPE) and late onset PE (LOPE), occurring before or after 34 weeks of gestation, respectively. EOPE is associated with poor trophoblast invasion and inadequate arterial remodeling and its pathophysiology is considered to be related to the placental development (129, 130). In physiological placentation, the maternal spiral arteries are invaded by extravillous trophoblast cells, which through a process named endovascular migration, gradually replace decidual ECs, leading to spiral artery remodeling (131–133) (**Figure 6**). When this process is impaired, it causes placental hypo-perfusion, tissue ischemia, vascular endothelium dysfunction, microangiopathic thrombosis, oxidative stress, and inflammatory response (134–136). In general, the cause of PE can be ascribed to an excessive maternal systemic inflammation in response to pregnancy through both innate and adaptive immune activation (137). Excessive inflammation during PE has also been confirmed through the measurement of procalcitonin, as a marker of sepsis or severe inflammation (138). The cause of the LOPE is more debatable, but the basal inflammatory state of the mother and the incompatibility between maternal supply and metabolic needs of the developing fetus (130) are considered key precipitating factors.

### Important Contribution of the Complement System in the Pathogenesis of Pre-Eclampsia

The C is an important component of the inflammatory process in PE (17, 18). Increased levels of C components and their activation products, including C1q, C3a, C5a and C5b-9 complex, in the circulation of PE patients as compared to normal pregnancy have been reported (17, 139–141), although the results in this field are quite discordant (141). C activation products have also been found in the urine of severe PE patients and are considered a marker of C-mediated renal damage (142). Elevated levels of the activation fragment Bb of the alternative pathway have been proposed as a predictive marker for the development of PE (143). Furthermore, increased C activation was demonstrated in several models of PE (144–146).

### Pre-Eclampsia: Diagnostic Criteria

Despite not sharing common pathological pathways, EOPE and LOPE are diagnosed by mutual clinical criteria (147). Uteroplacental under-perfusion, measured by the uterine artery pulsatility index (UtAPI), induces placental ischemia, which in turn, gives rise to oxidative-inflammation cascade activation, increased production of antiangiogenic factors, such as soluble fms-like tyrosine kinase 1 (sFlt-1) and soluble endoglin (sEng), and reduced production of angiogenic factors, such as placental growth factor (PlGF) (148, 149), both in the intrauterine environment and maternal endothelium. Interestingly, low maternal circulating levels of PlGF are measured prior to the clinical manifestation of PE and intrauterine growth restriction, as a marker of abnormal placentation (150). sFlt-1 is a soluble form of the vascular-endothelial growth factor (VEGF) and a receptor for PlGF. In the maternal circulation, sFlt-1 is able to bind to free VEGF and PlGF, reducing their bioavailability for membrane receptors (151). The use of a diagnostic test based on sFlt-1/PlGF ratio for the prediction of the short-term risk of PE may be a useful tool for patient management (152). In fact, with a sFlt-1/PlGF ratio of  ≤ 38 PE occurrence in the next week can be excluded with a negative predictive value of 99.3% (97.9% for ruling out within 2 weeks), whereas a ratio of >38 is associated to an enhanced risk of developing PE in the next 4 weeks (152, 153).

### Pre-Eclampsia in COVID-19 Pregnancy

Despite similar clinical manifestations between pregnant and non-pregnant women with COVID-19 (154), an increased rate of preterm delivery, PE, and cesarean section has been noticed in COVID-19 pregnant women (155–158). The INTERCOVID prospective longitudinal study showed an increased incidence of PE in pregnant women with COVID-19 (8.1%), as compared with non-diagnosed COVID-19 (4.4%), especially in nulliparous women; the association appeared to be independent of preexisting conditions and other risk factors, such as obesity, diabetes and hypertension (159). Mendoza et al. reported that a PE-like syndrome could be manifested in some pregnancies with critical COVID-19, according to the presence of severe pneumonia (160). The authors used the term PE-like syndrome to highlight some diverging aspects as compared to classical PE; in their cohort' the PE-like syndrome patients showed PE clinical signs and symptoms but normal parameters, such as sFlt-1/PlGF ratio, UtAPI and LDH <600 IU/l. This information could improve the management of these pregnancies since PE-like syndrome alone may not be considered as an obstetric indication for delivery (160).

Differential diagnosis in COVID-19 pregnant women developing hypertension, thrombocytopenia, proteinuria, as well as increased levels of liver enzymes, might be challenging, since misdiagnosis may occur due to COVID-19 and PE overlapping clinical features (147). In fact, a recent study suggested a two-fold increased risk of developing hypertensive disorders of pregnancy in patients who manifested COVID-19, especially early in their pregnancy (161); this is considered as a consequence of COVID-19-mediated modulation of placental ACE2 expression (162).

A common denominator in the pathophysiology of PE or PE-like syndrome and COVID-19 is the endothelial injury, due to disrupted placentation in PE (135, 163) and to directly or indirectly SARS-CoV-2-mediated damage to ECs in COVID-19 (71, 96, 164, 165). To clarify the extent of endothelial injury, the evaluation of two well-studied markers, such as the antiangiogenic factor sFlt-1 and the angiogenetic factor PlGF (166–168) can be useful. An imbalance between angiogenic and anti-angiogenic factors has been observed in COVID-19. Negro et al. indicated that an increase in sFlt-1 levels could be considered a good biomarker to predict survival and thrombotic events in COVID-19 patients (169). Smadja et al. considered PlGF increase as a relevant predictive factor for in‐hospital mortality to discriminate COVID‐19 severity (170), whereas Giardini et al. have used sFlt-1/PlGF ratio as a tool to stratify the severity of endothelial dysfunction (171). Interestingly, in PE-like syndrome, Mendoza et al. noted a normal sFlt-1/PlGF ratio and UtAPI assessment (160), suggesting normal values of sFlt-1/PlGF and UtAPI in COVID-19 patients with normal early phase of placental implantation, despite their symptomatic manifestations. Thus, an interplay between two different phenomena can be proposed in the clinical setting of SARS-CoV-2-infected obstetric patients at risk of developing COVID-19. At one hand, COVID-19 may mimic PE, particularly in early pregnancy; on the other hand, an already established PE may act as a risk factor for the development of severe or critical COVID-19. These two separate clinical conditions merit further investigation when clinical and epidemiological criteria indicate patients at risk of one or the other, or both conditions (172).

### Common Pathophysiology of Pre-Eclampsia and Severe COVID-19

Endothelial damage can be responsible for multi-organ dysfunction in both PE and COVID-19 (165, 173), as well as for an augmented risk of non-cardiogenic pulmonary oedema and venous thromboembolism (165). An increased hypercoagulable state characterizes PE women as compared to normal pregnancies, showing a rise in factor VIII, vWF, thrombin-antithrombin complex, D-dimers, soluble fibrin and TM levels (174). At the same time, the fibrinolytic system also plays an important role in PE, considering the significant increase in plasma plasminogen activator inhibitor type-1 (PAI-1) (174, 175). COVID-19 is also linked to a thrombogenic coagulopathy with a wide range of manifestations. COVID-19 patients commonly manifest mild thrombocytopenia (176) and increased D dimer levels (177) in accordance with disease severity, whereas other coagulation measurements are more variable (178, 179). Another feature shared between PE and COVID-19 patients is represented by an overall inflammatory microenvironment, characterized by an increase in serum and placental levels of pro-inflammatory and decrease of anti-inflammatory cytokines (180, 181).

In the context of endothelial damage, three common players have been proposed in PE and severe COVID-19 pathophysiological mechanisms: NETosis, anti-phospholipid antibodies (aPLAs) and α-1-antitrypsin (182). However, only the first two aspects concern an engagement of C.

There is an overwhelming case for the involvement of neutrophil extracellular traps (NETs) in immunothrombosis through several mechanisms: (a) NETs bind to vWF and recruit platelets; (b) NETs are able to trigger platelet activation; (c) NETs binding to TF provokes extrinsic pathway activation and thrombin generation; (d) Cleavage induced by neutrophil elastase and other neutrophil serine proteases inactivate anticoagulants, including TFPI and TM; and (e) NETs can directly support FXII activation mediated by platelet-derived polyphosphates (183). Interestingly, NETs have also been reported as contributors to PE pathogenesis, usually associated with maternal vasculitis, maternal-fetal interface hemorrhage and laminar decidual necrosis (184), and COVID-19-related endothelial damage and immunothrombosis through platelet-neutrophil interactions (185). NETs formation due to SARS-CoV-2 infection contains C3, factor B and properdin, triggering and stabilizing the alternative pathway convertase (67). An hyper-inflammatory immune state in response to abnormal neutrophil activation and NET formation, together with excessive or deregulated C activation, contributes to the well-documented clinical manifestations observed in severe COVID-19 (186). Moreover, NETs induce an excessive production of thrombin and the subsequent generation of C3a and C5a (108, 186). Hence, a feed-forward loop beginning with C activation may proceed with NETosis, consequently increased thrombin production, further stimulation of the C, and enhanced NET formation (67).

Another potential link between PE and COVID-19 is the presence of anti-phospholipid antibodies (aPLAs), since they have been indicated as an important risk factor for PE, especially EOPE (187). A recent study has reported elevated aPLA levels in nearly 52% of COVID-19 patients (188). In placenta, aPLAs promote platelet and EC activation, directly inducing procoagulant activity by interacting with factors of the coagulation pathway. This activity, however, was greatly reduced in C3 gene knock-out mice (189). Anti-β 2-glycoprotein-I, the primary pathogenic antibody in anti-phospholipid syndrome (190), is associated with an increased C activation (191), amplifying the production of other mediators of effector cell activation, including C3a, C5a, and MAC, with consequent thrombosis, tissue hypoxia, and inflammation within the placenta.

Interestingly, several findings suggest that genetic susceptibility may be involved in the dysregulation of C activation frequently observed in the progression to moderate/severe form of COVID-19 and in PE. Pathogenetic mutations or deletions in C factor and regulatory genes, which predispose to an increased C activation, have been identified both in pregnant women with PE/HELLP syndrome and in patient with COVID-19. C gene mutations were attributed to FH, MCP, FI and C3 (192–194). In COVID-19, gender differences were also observed in C-related variants, with women more genetically susceptible to C dysregulation (194).

## Therapeutic Considerations

Emerging evidence suggests that C is constantly activated in severe COVID-19 as well as PE; due to its ubiquity, potency and rapidity, it is reasonably considered as a potential drug target.

At present, the only effective treatment for PE remains parturition, since therapeutic approaches are mainly symptomatic. However, several preventative therapies are effective if administered early in the pregnancy (before 16 weeks of gestation): Low-dose Aspirin (LDA) and low-molecular weight heparin (LMWH) are the most common preventive treatments for PE. Both these drugs also modulate the C activity: LDA is able to down-regulate the levels of C3 and factor B expression in placenta (195, 196), whereas LMWH inhibits the activation of the alternative pathway and directly C5a (197–199). For its anti-thrombotic, anti-inflammatory, analgesic, and anti-pyretic effects, aspirin was also proposed in COVID-19 treatment, despite its clinical use for preventive or curative purposes has not been accepted yet (200–202). According to the beneficial anticoagulation effects of heparin observed in COVID-19 patients, with moderate or severe illness (203), pregnant women with severe COVID-19 should undergo thromboprophylaxis during hospitalization and at least until discharge (155). Moreover, in pregnant women with COVID-19, the use of steroids (dexamethasone followed by methylprednisolone) is recommended if clinically indicated (155). Several studies have reported the ability of methylprednisolone to inhibit C activation, particularly by acting on alternative pathway amplification (204, 205).

Other drugs are potentially used to prevent PE, especially targeting immunological conditions. For instance, it is well known that systemic lupus erythematosus (SLE) and anti-phospholipid Syndrome (APS) predispose to a higher risk of developing PE. Hydroxychloroquine (HCQ), an antimalarial drug, is often used in the treatment of SLE (206), resulting in a lower incidence of PE (196, 206). The mechanisms of action of HCQ on the C is debatable, so is its efficacy in the treatment of COVID-19. Most clinical trials have failed to demonstrate the efficacy of HCQ treatment in COVID-19 (207).

Recently, Lefkou et al. reported that the use of LDA+LMWH in addition to pravastatin (HMG-CoA reductase inhibitor) in APS-affected women at risk of developing PE reduced the onset of adverse outcomes, increasing placenta perfusion, reducing PE onset and improving neonatal outcomes (208). In murine studies, pravastatin inhibited C activation (C5a) by increasing the expression of C inhibitor, DAF (209).

The first C inhibitor to be approved for clinical trials was eculizumab, a monoclonal antibody able to block C5 and decrease C5a and C5b-9 formation (210). The use of eculizumab for C5 inhibition is a reasonable therapeutic option also in PE (196, 210), as suggested by a case report of a patient with severe PE/HELLP at 26 weeks of gestation, showing an improvement of woman and foetus clinical endpoints after eculizumab treatment (pregnancy was prolonged by 17 days, resulting in a reduction of neonatal morbidity).

There are currently 13 clinical studies investigating the effect of C inhibitors in the treatment of severe forms of COVID-19, but none of them include pregnant patients. These are mainly C3 and C5 inhibitors, including zilucoplan, AMY101, APL-9, eculizumab, and ravulizumab (211–214). In particular, eculizumab and ruxolitinib (JAK1/2 inhibitor) treatment resulted in clinical improvement within 3 days; this may be also due to their inhibitory effect on pathways responsible for local and hepatic C synthesis, such as NF-κB and STAT1/2 (215). Compstatin-based C3 inhibitor, AMY-101, showed a satisfactory efficacy in the treatment of severe COVID-19 pneumonia (63, 212). The lectin pathway inhibitor, narsoplimab, which exerts its activity by blocking MASP-2, has been shown to prevent EC damage and thrombotic microangiopathy; recovery and survival was observed in all COVID-19 patients treated with narsoplimab (216).

Despite the promising results in clinical trials, the potential use of both C3- and C5-targeted therapies in COVID-19 patients is undermined by some limitations as comparted to selective inhibitors (217). First, the relevance of C3 inhibitors is dependent on the timing: blocking the activation of all three C pathways may undesirably reduce viral clearance during the early disease, whilst they may be useful in advanced phases preventing uncontrolled C activation (77). On the other hand, blocking C5 activation prevents the proinflammatory and prothrombotic actions of the terminal products of the C cascade (C5a and C5b-9) activated by SARS-CoV-2, whilst preserving the activity of early C components involved in viral clearance and activation of the adaptive immune response (218); although, this exposes patients to the risk of developing other infections, especially bacterial (219).

Thus, a selective blockage of C5a (vilobelimab) or C5aR (avdoralimab) could be more beneficial since it preserves the formation of C5b-9 complex as a crucial player in pathogen elimination (220, 221). The blockage of C5a-C5aR1 axis limits the infiltration of myeloid cells in damaged organs, hampers the production of pro-thrombotic factors by immune cells, platelets, and ECs, as well as prevents the excessive lung inflammation and endotheliitis associated with acute respiratory distress syndrome in patients with COVID-19 (58, 64, 222).

Another interesting therapeutic option under clinical investigation is the recombinant human C1–INH conestat alfa, which yielded encouraging results (223). Due to its multifaceted inhibitory action, C1–INH prevents all three C pathways’ activation (36–38, 113) and inhibits components of the coagulation cascade, plasmin and kallikrein, reducing C-driven inflammation and coagulation (113).

## Conclusions and Perspectives

A link between PE and COVID-19 has clearly emerged due to overarching pathophysiological mechanisms involved, which are triggered by an interplay between C and coagulation. Mothers or women who have recovered from COVID-19 need to be monitored closely during subsequent pregnancies for immune deviations. A very important question that is not yet solved and described in the use of C inhibitors for COVID-19 patients is the definition of the right window of opportunity for treatment. In these high-risk patients, with a major genetic susceptibility associated to complement polymorphisms, genetic testing might be an important tool aiming at intensified monitoring and early initiation of specific treatment with C inhibitors. Furthermore, the exact role of the C in severe COVID-19 development is yet to be clarified, which includes C-driven cytokines and viral protein activation of C–coagulation crosstalk.

## Author Contributions

CA, AM, AB, AA, GR, UK, and RB reviewed the literature and wrote sections of the review article. CA and AA created figures. UK critically reviewed the entire manuscript. All authors contributed to the article and approved the submitted version.

## Funding

This research was supported by Ferring COVID-19 Investigational Grant (GRAVISAR to RB) and by the Institute for Maternal and Child Health, IRCCS Burlo Garofolo, Trieste, Italy (RC24/19 to GR and 09/21 to CA). The funder Ferring Pharmaceuticals was not involved in the study design, collection, analysis, interpretation of data, the writing of this article or the decision to submit it for publication.

## Conflict of Interest

The authors declare that the research was conducted in the absence of any commercial or financial relationships that could be construed as a potential conflict of interest.

## Publisher’s Note

All claims expressed in this article are solely those of the authors and do not necessarily represent those of their affiliated organizations, or those of the publisher, the editors and the reviewers. Any product that may be evaluated in this article, or claim that may be made by its manufacturer, is not guaranteed or endorsed by the publisher.

## Glossary

**Table:**

ACE2	angiotensin-converting enzyme 2
aPLAs	anti-phospholipid antibodies
APS	anti-phospholipid syndrome
BK	bradykinin
C	complement system
C1-INH	C1-inhibitor
COVID-19	coronavirus disease 2019
CR1	complement receptor 1
DAF	decay accelerating factor
DAMPs	damage-associated molecular patterns
EC	endothelial cell
EMMPRIN	extracellular matrix metalloproteinase inducer
EOPE	early onset pre-eclampsia
F	factor
HCQ	hydroxychloroquine
HELLP	hemolysis, elevated liver enzymes and low platelets
iTCC	inactive terminal complement complex
LDA	low-dose aspirin
LMWH	low-molecular weight heparin
LOPE	late onset pre-eclampsia
MAC	membrane attack complex
MASPs	MBL-associated serine proteases
MBL	mannan-binding lectin
MCP	membrane cofactor protein
MERS	middle east respiratory syndrome
NETs	neutrophil extracellular traps
PAI-1	plasminogen activator inhibitor type-1
PAMPs	pathogen-associated molecular patterns
PARs	protease-activated receptors
PE	pre-eclampsia
PlGF	placental growth factor
RAAS	renin-angiotensin-aldosterone system
ROS	reactive oxygen species
SARS-CoV-2	severe acute respiratory syndrome coronavirus 2
sEng	soluble endoglin
sFlt-1	soluble fms-like tyrosine kinase 1
SLE	systemic lupus erythematosus
vWF	von Willebrand factor
TAFI	thrombin-activable fibrinolysis inhibitor
TCC	terminal complement complex
TF	tissue factor
TFPI	tissue factor pathway inhibitor
TM	thrombomodulin
TMPRSS2	transmembrane serine protease 2
UtAPI	uterine artery pulsatility index
VEGF	vascular-endothelial growth factor
